# Experiences of Ageism Among Older Adults Registered with a Family Health Centre: A Mixed-Methods Research Study

**DOI:** 10.3390/healthcare14060801

**Published:** 2026-03-21

**Authors:** Zeliha Yelda Özer, Yusuf Kemal Arslan, Çağla Okyar, Çiğdem Gereklioğlu

**Affiliations:** 1 Department of Family Medicine, Faculty of Medicine, Çukurova University, 01330 Adana, Türkiye; 2 Department of Biostatistics, Faculty of Medicine, Çukurova University, 01330 Adana, Türkiye; ykarslan@gmail.com; 3 Department of Medical Education and Informatics, Faculty of Medicine, Çukurova University, 01330 Adana, Türkiye; cokyar@cu.edu.tr; 4 Department of Family Medicine, Faculty of Medicine, Başkent University, 01120 Adana, Türkiye; gereklioglucigdem@hotmail.com

**Keywords:** ageism, primary health care, aged

## Abstract

**Background/Aim**: This study aimed to describe experiences of ageism and to explore related perceptions among older adults registered at an Education Family Health Centre (EFHC) in Türkiye. **Methods**: This mixed-methods study was conducted with 83 older adults registered with the education family health center (EFHC). In the quantitative part of the study, surveys (the Sociodemographic data form and the Ageism Survey, which evaluates the negative aspects of ageism) were administered face-to-face. In the qualitative section, four semi-structured questions about ageism were asked by phone. A descriptive cross-sectional survey design was used in the quantitative component of the study, and thematic analysis in the qualitative part. **Results**: The mean age of the participants was 69.9 ± 4.8 years. According to Ageism Survey, 79.6% of participants gave a score higher than 0 and indicated that they had experienced age discrimination at least once. Eight participants completed the qualitative section, and three themes were identified: (1) Self-directed ageism, (2) Increasing health problems and social isolation with age, and (3) Ageism. **Conclusions**: In this EFHC-based sample, the majority of participants reported at least one experience of ageism. Social awareness about ageism needs to be increased. Educational interventions can be planned at the micro, meso, and macro levels to raise awareness and combat ageism. Family physicians who serve with a holistic approach can play a pivotal role in this regard.

## 1. Introduction

Ageism was first defined by Robert N. Butler in 1969 as the prejudice of one age group against another age group [1]. In 1975, Butler defined ageism as not only prejudice but also the systematic stereotyping and discrimination of people because of their age, similar to racism and sexism [2]. However, ageism differs from racism and sexism in that all people age. While Butler draws attention only to harmful discrimination against older persons in his definition of ageism, some studies also mention positive prejudice, stereotyping, and discrimination against older persons [3]. Compassionate stereotypes and pitying positive definitions are examples [4]. Positive discrimination can result in positive outcomes, but it can also result in the overcaring, belittling, and passivization of older people. Another positive stereotype, sageism, is that older people are wiser because they have lived longer than younger people [5].

Ageism can be implicit (unconscious) or explicit (conscious) and can be expressed at the micro (individual), meso (social network), or macro (institutional and cultural) levels [2]. At the micro level, ageism is addressed via a three-dimensional approach, namely, uniformization (cognitive dimension), prejudice (intellectual dimension), and discrimination (behavioral dimension) [6]. Another individual case that needs to be considered in ageism is self-directed age discrimination, which refers to stereotyping, prejudice, and discrimination against oneself based on age or perceived age [7].

Globally, the population aged 65 and over is growing faster than any other age group. By 2050, the number of people is expected to double worldwide [8]. In Türkiye, the proportion of older persons in the total population reached 10.2% in 2023 [9].

Along with the ageing population, problems specific to older persons are naturally increasing, and ageism is one of these problems. Ageism is associated with earlier death (by 7.5 years), poorer physical and mental health, and slower recovery from disability in older age [10]. Although ageism has long been recognized as the most common form of discrimination, the paucity of research in this area is striking [11].

The Education Family Health Centre (EFHC) is a primary healthcare unit affiliated with a university hospital, providing comprehensive preventive and curative services to a defined community population. Such centers represent an important setting for investigating social determinants of health, including age-related discrimination, as they serve older adults with diverse socioeconomic and health backgrounds.

Despite the growing global recognition of ageism as a social determinant of health, evidence from middle-income countries remains limited. In Türkiye, available studies have largely focused on age-related attitudes among healthcare students or physicians rather than directly on older adults’ lived experiences of ageism. Therefore, evidence on perceived ageism among community-dwelling older adults, particularly in primary care settings, remains limited [12,13]. Understanding these experiences is important for identifying vulnerable groups and informing age-friendly healthcare practices.

Previous studies have suggested that ageism experiences among older adults may be influenced by various socio-demographic and health-related factors, including age, gender, educational level, socioeconomic status, living arrangements, and health status [14,15]. These factors may shape both the perception and the experience of age-related discrimination in everyday life and in healthcare settings. Therefore, examining these characteristics may help identify groups that are more vulnerable to ageism and inform age-friendly primary care practices.

Despite increasing global attention to ageism, empirical evidence from Türkiye remains limited, particularly in primary healthcare settings. By examining perceived ageism among older adults registered in a university-affiliated primary care center, this study seeks to contribute to the limited national literature and provide insights relevant for developing age-friendly healthcare practices.

This study aimed to investigate the proportion of ageism and related factors among older adults registered with EFHC and to inform age-friendly primary care practices by identifying commonly reported forms of ageism.

## 2. Methods

Ethical approval for this mixed-methods study was obtained from the Academic Board of the Department of Family Medicine, Faculty of Medicine, Çukurova University, and the Ethics Committee for Non-Interventional Clinical Research at Çukurova University (3 June 2022; decision no. 123). All procedures involving human participants were conducted in accordance with the ethical standards of the institutional and national research committees and with the principles of the Declaration of Helsinki and its later amendments.

Written informed consent was obtained from all participants prior to participation. Participants did not receive any financial or material compensation for their participation in the study. Permission to use the Turkish version of the Ageism Survey (AS) was obtained from the investigator who validated the scale.

### 2.1. Study Design

This study employed an explanatory sequential mixed-methods design, in which quantitative data collection and analysis were followed by a qualitative phase aimed at providing deeper insight into participants’ experiences and perceptions related to ageism.

The quantitative component used a cross-sectional survey design conducted in a university-affiliated primary care center. The qualitative component consisted of semi-structured in-depth interviews, and thematic analysis was used to explore participants’ experiences and perceptions of aging and ageism.

### 2.2. Population, Sample/Study Group, and Data Collection

The study was conducted with 83 participants aged 65 years and above between 16 June 2022 and 13 July 2023. The study population consisted of older adults registered in Çukurova University EFHC, and the sample consisted of older adults who agreed to participate, resulting in a non-probabilistic convenience sample. No a priori sample size calculation was performed because the study aimed to reach all eligible registered older adults during the data collection period. Patients who were registered with the EFHC, 65 years and older, being able to speak Turkish, who volunteered for participation, having no severe neurological and/or psychiatric disorder that may hinder participation and/or communication were included in the study. Those who had a diagnosis of dementia and were unwilling to participate in the study were excluded.

As the study was designed as an exploratory cross-sectional analysis, no formal a priori power calculation was performed. However, the achieved sample size was considered adequate to explore associations between perceived ageism and selected socio-demographic and health-related characteristics.

**Figure 1:**
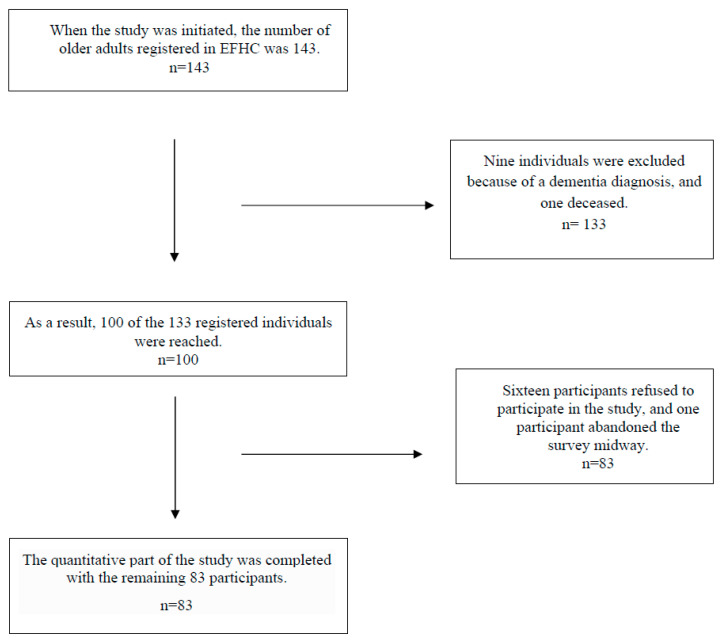
The flowchart of participants in the quantitative part of the study.

Data were collected via the face-to-face survey method. In the quantitative part of the study, a sociodemographic data form consisting of 15 questions and an AS was used. When the study was initiated, the number of older adults registered in EFHC was 143. Nine individuals were excluded because of a dementia diagnosis, and one deceased. As a result, 100 of the 133 registered individuals were reached, and 16 of them refused to participate in the study, and one left the survey unfinished. The quantitative part of the study was completed with the remaining 83 participants. The flowchart of participants in the quantitative part of the study is presented in Figure 1.

The study population consisted of older adults aged 65 years and above who were registered at the Education Family Health Centre (EFHC) affiliated with Çukurova University.

At the time the study was initiated, a total of 143 older adults aged 65 years and over were registered in three Family Medicine Units (FMUs) of the Education Family Health Centre: 33 (23.1%) in unit 047, 15 (10.5%) in unit 051, and 95 (66.4%) in unit 056. During the quantitative data collection process, 9 individuals with a diagnosis of dementia and 1 deceased individual were excluded from the sampling frame. Thus, 133 older adults constituted the target population for the study.

However, during the data collection period, only 100 of these 133 eligible individuals could be reached. Among the individuals reached, 16 declined to participate (8 stated that they did not have time, 2 reported that they did not feel well, 3 stated that they wanted to return home as soon as possible, 2 reported that they did not want to answer the questions, and 1 did not provide a reason), and one participant left the questionnaire incomplete due to emotional distress related to losses experienced during the earthquake. Consequently, the quantitative phase of the study was completed with 83 participants.

Data for the quantitative component were collected through face-to-face surveys conducted between 16 June 2022 and 13 July 2023. The survey consisted of a sociodemographic data form and the Ageism Survey.

For the qualitative phase of the study, in-depth telephone interviews were conducted with 8 participants selected from the study population. The participant flow diagram is presented in Figure 1.

#### Qualitative Sampling

For the qualitative component, participants from the quantitative phase were contacted by telephone and invited to participate in in-depth interviews. A total of 83 participants who had completed the quantitative survey were approached. However, only 11 individuals could be reached, likely due to population displacement following the 6 February 2023 earthquakes in Türkiye, which severely affected the area surrounding the Education Family Health Centre.

Among those reached, three participants declined participation, citing reasons such as hearing difficulties, relocation, or unwillingness to discuss the topic further. Ultimately, eight participants agreed to participate in the qualitative interviews.

Participants were coded using the letter “P” followed by a number indicating the order of participation (e.g., P1, P2). Although no substantially new codes emerged after the fourth interview, interviews were continued to confirm thematic adequacy. All qualitative interviews were conducted individually on a one-to-one basis via telephone by the same researcher (ZYÖ). The average duration of the interviews was approximately 14 min (range: 5–21 min).

### 2.3. Data Collection Instruments

#### 2.3.1. Sociodemographic Data Form

A structured form consisting of 15 items was developed by the researchers based on the literature. The questionnaire collected information on participants’ demographic and socioeconomic characteristics, including age, gender, educational status, past and present employment status, occupation, income status, marital status, number of children and living arrangements.

Participants were also asked whether they had any physician-diagnosed chronic diseases, including hypertension, diabetes mellitus, cardiovascular disease, depression, or other chronic conditions.

#### 2.3.2. Ageism Survey

Perceived ageism was assessed using the Ageism Survey (AS) developed by Palmore (2001) [16]. The scale consists of 20 items assessing experiences of age-related discrimination.

Each item is scored on a three-point Likert scale: 0 = never, 1 = once, 2 = more than once. Total scores range from 0 to 40, with higher scores indicating more frequent experiences of age-related discrimination.

An example item from the scale is:

*“Have you ever been treated with less respect than other people because of your age?”*

The Turkish version of the scale was validated by Erol et al. (2016), with a Cronbach’s alpha of 0.86 [17].

The full Ageism Survey questionnaire is provided as Supplementary Materials File S1.

### 2.4. Statistical Analysis

#### 2.4.1. Quantitative Data Analysis

Continuous variables were summarized as median and interquartile range (IQR) due to non-normal data distribution. Categorical variables were expressed as frequencies and percentages. The Kolmogorov–Smirnov test was used to assess normality. As the data showed non-normal distribution, nonparametric tests were used. Group comparisons were performed using Mann–Whitney U test for two-group comparisons and Kruskal–Wallis test for comparisons involving more than two groups. When the Kruskal–Wallis test indicated statistical significance, Dunn’s post hoc test was performed. Test statistics for the Mann–Whitney and Kruskal–Wallis tests are reported alongside *p*-values. All statistical analyses were performed using IBM SPSS Statistics version 20.0 (IBM Corp., Armonk, NY, USA). Statistical significance was set at *p* < 0.05.

#### 2.4.2. Qualitative Data Analysis

The qualitative phase was designed to provide contextual understanding of patterns observed in the quantitative findings.

Qualitative data were collected through semi-structured interviews consisting of four open-ended questions exploring participants’ experiences and perceptions related to aging and ageism (Table 1).

All interviews were conducted individually via telephone. Interviews were transcribed verbatim and analyzed using inductive thematic analysis. Transcripts were first read independently by the researchers and then discussed collectively during team meetings. Coding was conducted through an inductive approach, allowing themes and subthemes to emerge from the data rather than being predetermined. During analysis, researchers engaged in iterative discussions and reflective interpretation of participants’ statements. The qualitative component was conducted and reported in accordance with the Consolidated Criteria for Reporting Qualitative Research (COREQ) guidelines.

The COREQ 32-item checklist is provided as Supplementary Materials File S2.

## 3. Results

A total of 83 older adults participated in the quantitative component of the study. The mean age of the participants was 69.9 ± 4.8 years (range, 65–90 years). Participants’ sociodemographic and health-related characteristics are presented in Table 2.

When responses to the Ageism Survey (AS) were evaluated, 17 participants (20.4%) reported no experience of age-related discrimination (score = 0), whereas 66 participants (79.6%) reported at least one ageism-related experience. The mean AS total score was 4.3 ± 4.6, with an observed range of 0–20, although the theoretical range of the scale is 0–40. This finding suggests that while many participants reported at least one ageism-related experience, the overall frequency of such experiences was relatively low in this sample. The fact that no participant scored above 20 indicates that repeated or multiple ageism-related experiences were uncommon.

The distribution of AS total scores according to participant characteristics is shown in Table 3. Given the non-normal distribution of the data, AS scores were summarized as median and interquartile range (IQR), and non-parametric tests were used for group comparisons.

AS total scores differed significantly according to educational level and profession. Participants with university education had lower AS scores than illiterate participants (*p* = 0.006) and those with primary school education (*p* = 0.003). In addition, officers had lower AS scores than workers (*p* = 0.028) and homemakers (*p* = 0.005). A significant difference was also observed according to past employment status, with participants who had previously worked having lower AS scores than those who had never worked (*p* = 0.044). No statistically significant differences were found according to sex, current employment status, working conditions, income status, marital status, number of children, living arrangement, self-reported discomfort, or chronic disease status.

Because these analyses were exploratory and based on bivariate comparisons in a relatively small convenience sample, the observed associations should be interpreted cautiously.

In addition to total AS scores, selected AS items were explored according to participant characteristics. Participants who reported discomfort were less likely to report being told jokes that made fun of older people (*p* = 0.034), being called nicknames related to age (*p* = 0.043), and receiving less dignity and respect because of their age (*p* = 0.037). With respect to educational status, no university graduate reported receiving less dignity and respect because of age, whereas such experiences were reported among participants with lower educational attainment (*p* = 0.033). Similarly, none of the participants with university education reported having been denied medical treatment because of age, whereas this experience was reported by some participants with lower educational levels. Given the number of comparisons performed, these item-level findings should be considered exploratory.

For the qualitative component, eight participants took part in individual telephone interviews. Their demographic characteristics and AS scores are summarized in Table 4.

Interview duration ranged from 5 to 21 min, with a mean duration of 14 min. A total of 27 codes, four categories, and three themes were identified. To improve conceptual clarity, themes related to general experiences of aging were distinguished from those directly reflecting ageism. The three themes were: (1) self-directed age-related stigma, (2) perceived age-related limitations, and (3) interpersonal experiences of ageism. The code tree is presented in the Supplementary Materials File S3.

### 3.1. Theme 1: Self-Directed Age-Related Stigma

This theme reflects participants’ internalized negative perceptions about their own aging and loss of value over time.

#### Subtheme: Age-Related Self-Stigma

Some participants described aging in a self-devaluing manner, suggesting internalized age-related stereotypes.

*“You are not like when you were young. You are old; you have been cast aside”*.

(P1)

*“You are finished. In youth, you are like fire, then you turn into ashes”*.

(P2)

*“You are past your prime”*.

(P7)

### 3.2. Theme 2: Perceived Age-Related Limitations

This theme captures participants’ descriptions of physical, functional, and social changes associated with aging. These accounts reflect perceptions of aging rather than direct experiences of ageism.

#### Subtheme: Physical Changes and Functional Restriction

Participants frequently described pain, fatigue, reduced mobility, sleep disturbance, and limitations in daily or social activities.

*“I have pain. I have no sleep pattern. If I go to bed at 12 at night, I get up at 5 in the morning”*.

(P7)

*“We lost sleep. We used to walk 10 km; now we walk 2 or 3 km, and of course, we ache all over. We get tired”*.

(P7)

*“Of course, you have to limit social activities. There are physical changes in terms of health; I get tired earlier, I get tired faster than before”*.

(P6)

*“Not being able to work like before. Fatigue, we get tired quickly”*.

(P4)

*“We cannot move freely like we used to. We can do our own work, but not as well as before. In the past, we used to do a job in a rush; everything used to come to our minds like clockwork. Now sometimes we forget”*.

(P4)

### 3.3. Theme 3: Interpersonal Experiences of Ageism

This theme includes participants’ reports of disrespectful, excluding, or dismissive behaviors from others that they attributed to their age.

#### Subtheme, Negative Behavior Toward Older Adults

Participants described experiences of being ignored, disrespected, mocked, or looked down upon in daily life, particularly in public spaces.

*“There is age discrimination; young people do not recognize us. They do not sit next to us; they do not come. Excuse me, I stand with my wife as if we are standing in a coop. Young people do not converse with us”*.

(P5)

*“Especially young people are degenerating in everything, but there is no respect. I am complaining in that respect. I take the bus because my neighborhood is home to many students. They ignore you or make fun of you”*.

(P6)

*“I am complaining about young people. They used to sleep on the bus; now, they are using their cell phones to avoid being seen. Disrespect is at its peak among young people”*.

(P6)

*“Yes, especially on the bus, I have a 65-year-old card. When I get on the bus, the driver and the citizens glare at me. The state pays for us. They look down on us”*.

(P8)

The qualitative findings provided contextual insight into the quantitative results by illustrating how age-related disrespect, exclusion, and negative labeling were experienced in everyday life, while also showing that some participant narratives reflected broader perceptions of aging rather than ageism alone.

## 4. Discussion

In this study, 79.6% of participants reported experiencing at least one age-related discriminatory event in the survey. However, the overall frequency of such experiences appeared relatively low, as reflected by the mean Ageism Survey score. These findings suggest that while isolated experiences of age-related discrimination may occur among older adults, repeated or pervasive forms of ageism were less frequently reported in this sample. Similar patterns of age-related discriminatory experiences have been reported in different populations worldwide [18].

Another striking observation during the face-to-face survey process was that many participants were unfamiliar with the concept of ageism itself. This may reflect limited public awareness of age-based discrimination in the local context. Health policies addressing ageism in Türkiye remain relatively limited. Increasing awareness at both the individual and societal levels may therefore represent an important step toward recognizing ageism as a social determinant of health. Primary care settings may play a key role in this regard. Family physicians are often the first point of contact within the health system, and through preventive counseling and community-based education, they may contribute to raising awareness about ageism and its consequences.

For example, one participant described being stared at by other passengers when boarding public transportation using a transportation card provided for older adults. This quotation illustrates how subtle forms of social stigma may occur in everyday life and highlights the need to address ageism at both micro and macro levels. Although some measures have been implemented at the policy level, the lack of interventions addressing everyday social interactions may restrict the social participation and mobility of older adults.

In our study, one of the most commonly reported forms of ageism was “A joke was told making fun of old people.” However, exposure to this and other forms of ageism, such as being called an insulting name related to age or receiving less dignity and respect because of being older, appeared to be less frequent among participants with health problems or higher levels of education. Cultural norms may partially explain these findings [19]. Health and educational status are important factors that influence perceptions, attitudes, and behaviors toward oneself and others in Turkish society [20]. Cultural norms may shape both exposure to and interpretation of age-related experiences. In many contexts within Turkish culture, individuals with health problems may receive additional care and attention, while those with higher educational status may be treated with greater respect. Culture is recognized as one of the key factors influencing ageism not only in Türkiye but also worldwide [21].

Among occupational groups, officers reported fewer experiences of ageism compared with workers and homemakers. This finding may be related to socioeconomic differences between occupational groups, including educational level and economic resources. Previous research has shown that social vulnerability may increase the risk of experiencing age-related discrimination [22].

Similarly, individuals with higher levels of education reported fewer experiences related to healthcare discrimination, such as being denied medical treatment because of age. Studies in the literature support the relationship between educational status and perceived healthcare discrimination [23]. However, the mechanisms underlying age-related bias in healthcare settings are complex. Physicians’ knowledge, expectations, and attitudes regarding ageing may influence their behavior toward older patients when providing healthcare services [24]. In addition, how individuals perceive and interpret their own ageing experiences may shape their perception of discrimination in healthcare contexts [25]. For this reason, incorporating educational content addressing ageism and ageing into medical education curricula may contribute to increasing awareness among healthcare professionals.

The mixed-methods design of the present study allowed us to explore ageism from multiple perspectives. In the qualitative component, participants described not only interpersonal experiences of age discrimination but also negative perceptions regarding their own ageing process. These narratives reflect elements of self-directed ageism, in which individuals internalize societal stereotypes about ageing and apply them to themselves [26]. Previous studies have suggested that self-directed ageism may be associated with adverse health outcomes, including increased mortality and morbidity [27]. In addition, negative perceptions of ageing have been linked to inflammatory processes and other physiological stress responses [28].

However, the qualitative findings also indicated that some experiences described by participants were related not only to ageism but also to broader perceptions of ageing and physical limitations associated with increasing age. Therefore, in the revised analysis, we distinguished between interpersonal ageism, self-directed ageism, and perceived age-related limitations in order to avoid conceptual confusion. Age-related physiological changes and social withdrawal may contribute to social isolation among older adults, which has been associated with increased risks of depression, dementia, and cardiovascular disease [29]. Addressing both external and internalized forms of ageism may therefore represent an important component of promoting healthy ageing.

One of the strengths of this study is the use of a mixed-methods design that combines quantitative and qualitative approaches. The quantitative component provided information about the frequency of age-related experiences, while the qualitative interviews allowed a deeper understanding of how older adults interpret these experiences in their daily lives. Another strength is that the data were collected within a primary care setting, which provides services to individuals across the life course.

Several limitations should also be considered. The most important limitation of this study was the earthquake disaster that occurred in Türkiye on 6 February 2023, which interrupted the data collection process and limited our ability to reach all potential participants. In addition, the single-center design and convenience sampling approach limit the generalizability of the findings. Future studies using probability-based sampling methods and multicenter designs would help provide a more comprehensive understanding of ageism among older adults.

### Future Perspectives

Future research should investigate ageism in larger, multicenter, and more socioeconomically diverse samples of older adults in Türkiye. In addition to conventional sociodemographic variables, an intersectional approach may provide a deeper understanding of how ageism is experienced and internalized. Factors such as sexual orientation, gender, educational disadvantage, socioeconomic vulnerability, living arrangements, and health status may interact to shape both perceived ageism and self-directed ageism. Further mixed-methods and longitudinal studies are needed to clarify these complex relationships and to guide the development of inclusive, age-friendly health and social care policies.

## 5. Conclusions

This study suggests that experiences of age-related discrimination are present among older adults in a primary care setting, although the overall frequency of such experiences appears relatively low. Educational and occupational factors may influence how ageism is perceived and experienced. The qualitative findings further indicate that both interpersonal ageism and self-directed ageism may shape how older adults interpret ageing and their position within society. Considering ageism as a social determinant of health may help guide future health policies and public health initiatives. Family medicine plays a key role as the first point of contact within the healthcare system and may contribute to the prevention, early recognition, and awareness of ageism. Educational initiatives targeting healthcare professionals, institutions, and the broader community may help reduce age-related stereotypes and improve the wellbeing of older adults. Given the rapidly ageing global population, further large-scale, multicentre studies are needed to better understand the complex social, cultural, and structural factors influencing ageism.

## Figures and Tables

**Table 1:** Semi-structured open-ended questions.

(1) What are your thoughts on ageing?
(2) What kind of changes do you think have happened in your life with getting older?
(3) What do you think is the change in your life that affects you the most as you get older?
(4) What do you think about ageism?

**Table 2:** Sociodemographic and health-related characteristics of the participants (n = 83).

Characteristic	n	%
Sex		
Female	35	42.2
Male	48	57.8
Education status		
Illiterate	16	19.3
Primary education	33	39.8
Secondary education	17	20.5
University and above	17	20.5
Past employment status		
Did not work	14	16.9
Worked	69	83.1
Current employment status		
No	72	86.7
Yes	11	13.3
Profession		
Housewife	17	20.5
Officer	17	20.5
Worker	32	38.6
Self-employed	17	20.5
Type of occupational workload		
Mainly physical	9	10.8
Mainly mental	17	20.5
Both physical and mental	57	68.7
Income status		
Income lower than expenses	56	67.5
Income equal to expenses	23	27.7
Income higher than expenses	4	4.8
Marital status		
Single	4	4.8
Married	56	67.5
Divorced	10	12.0
Widowed	13	15.7
Number of children		
No children	5	6.0
One child	11	13.3
More than one child	67	80.7
Living arrangement		
With spouse	43	51.8
With spouse and children	13	15.7
With children	11	13.3
Alone	16	19.3
Self-Reported Health Complaint		
No	13	15.7
Yes	70	84.3
Physician-diagnosed chronic disease (e.g., hypertension, diabetes mellitus, coronary artery disease, depression)		
No	7	8.4
Yes	76	91.6

Data are presented as n (%).

**Table 3:** Ageism Survey (AS) total scores according to sociodemographic and health characteristics.

	Median (IQR)	Test Statistic (Mann–Whitney U/Kruskal–Wallis H)	*p*
Sex			
Female	4 (2–7)	2.619	0.106
Male	2 (0–5.5)		
Education status			
Illiterate	4 (2–6.5)	13.688	0.033
Primary education	4 (2–7)		
Secondary education	3 (1–7)		
University and above	1 (0–2)		
Past employment status			
Did not work	5 (3–7)	4.070	0.044
Worked	2 (1–5)		
Current employment status			
No	3 (1–6.5)	1.437	0.231
Yes	1 (0–4)		
Profession			
Housewife	5 (3–7)	10.741	0.013
Officer	1 (0–2)		
Worker	3 (1.5–7.5)		
Self-employed	2 (0–4)		
Type of occupational workload			
Mainly physical	2 (2–7)	2.541	0.281
Mainly mental	2 (1–4)		
Both physical and mental	3 (1–7)		
Income status			
Income lower than expenses	3 (1–6)	1.284	0.526
Income equal to expenses	4 (0–9)		
Income higher than expenses	1.5 (0.5–3)		
Marital status			
Single	2.5 (1–10)	0.687	0.876
Married	2.5 (0.5–6.5)		
Divorced	2.5 (2–5)		
Widowed	4 (2–6)		
Number of children			
No children	3 (2–6)	3.008	0.223
One child	3 (1–4)		
More than one child	2 (0–20)		
Living arrangement			
With spouse	3 (0–6)	1.678	0.642
With spouse and children	2 (1–7)		
With children	4 (2–7)		
Alone	2.5 (1–5)		
Self-Reported Health Complaint			
No	2 (0–7)	0.295	0.587
Yes	3 (1–6)		
Physician-diagnosed chronic disease (e.g., hypertension, diabetes mellitus, coronary artery disease, depression)			
No	2 (1–5)	0.307	0.580
Yes	3 (1–6.5)		

Data are presented as median (interquartile range). Group comparisons were performed using the Mann–Whitney U test for two-group comparisons and the Kruskal–Wallis test for comparisons involving three or more groups. The test statistics presented correspond to the Mann–Whitney U statistic or the Kruskal–Wallis H statistic, as appropriate.

**Table 4:** Sociodemographic characteristics and Ageism Survey (AS) scores of participants included in the qualitative interviews (n = 8).

Participant Code	Sex	Age (years)	Educational Status	Profession	AS Score
P1	Female	68	Primary education	Housewife	5
P2	Male	68	Secondary education	Retired worker	3
P3	Male	73	University education	Retired officer	4
P4	Female	68	Primary education	Housewife	7
P5	Male	85	Primary education	Retired worker	6
P6	Female	69	Secondary education	Retired worker	3
P7	Male	67	Secondary education	Worker	0
P8	Male	66	Primary education	Self-Employed	18

AS: Ageism Survey. Scores range from 0 to 40, with higher scores indicating more frequent experiences of age-related discrimination.

## Data Availability

The data are not publicly available due to privacy considerations. Therefore, they are available from the corresponding author upon reasonable request.

## Supplementary Materials

The following supporting information can be downloaded at: https://www.mdpi.com/article/10.3390/healthcare14060801/s1, File S1: The Ageism Survey; File S2: COREQ 32-item checklist; File S3: Code Tree.
