# Supplementary material for: Experiences of Ageism Among Older Adults Registered with a Family Health Centre: A Mixed-Methods Research Study
Source: Healthcare (Basel). 2026 Mar 21;14(6):801. doi: 10.3390/healthcare14060801 (PMC13026802; doi:10.3390/healthcare14060801)
Supplement: Supplementary file 1 [file healthcare-14-00801-s001.zip › File S1.pdf]

# Appendix

## *The Ageism Survey*[a]

Please put a number in the blank that shows how often you have experienced that event: Never = 0; Once = 1; More than once = 2. ("Age" means older age.)

______ 1. I was told a joke that pokes fun at old people.

______ 2. I was sent a birthday card that pokes fun at old people.

______ 3. I was ignored or not taken seriously because of my age.

______ 4. I was called an insulting name related to my age.

______ 5. I was patronized or "talked down to" because of my age.

______ 6. I was refused rental housing because of my age.

______ 7. I had difficulty getting a loan because of my age.

______ 8. I was denied a position of leadership because of my age.

______ 9. I was rejected as unattractive because of my age.

______10. I was treated with less dignity and respect because of my age.

______11. A waiter or waitress ignored me because of my age.

______12. A doctor or nurse assumed my ailments were caused by my age.

______13. I was denied medical treatment because of my age.

______14. I was denied employment because of my age.

______15. I was denied promotion because of my age.

______16. Someone assumed I could not hear well because of my age.

______17. Someone assumed I could not understand because of my age.

______18. Someone told me, "You're too old for that."

______19. My house was vandalized because of my age.

______20. I was victimized by a criminal because of my age.

Please write in your age: ____

Please check: Male ____ or Female ____

What is the highest grade in school that you completed? ____

[a]Survey © Copyright 2000 by Erdman Palmore.
