# Supplementary material for: Experiences of Ageism Among Older Adults Registered with a Family Health Centre: A Mixed-Methods Research Study
Source: Healthcare (Basel). 2026 Mar 21;14(6):801. doi: 10.3390/healthcare14060801 (PMC13026802; doi:10.3390/healthcare14060801)
Supplement: Supplementary file 1 [file healthcare-14-00801-s001.zip › File S2.pdf]

Supplementary File S2. COREQ 32-item checklist

No Item	Guide questions/description	
**Domain 1: Research team and reflexivity**		
Personal Characteristics		
1. Interviewer/facilitator	Which author/s conducted the interview or focus group?	All interviews were conducted by Zeliha Yelda Özer (ZYÖ)
2. Credentials	What were the researcher's credentials? E.g. PhD, MD	Zeliha Yelda Özer, Family Physician, MD. Yusuf Kemal Arslan (YKA), Biostatistician, PhD. Çağla Okyar (ÇO), General Practitioner MD. Çiğdem Gereklioğlu (ÇG), Family Physician, MD.
3. Occupation	What was their occupation at the time of the study?	All researchers were faculty members at the medical school
4. Gender	Was the researcher male or female?	ZYÖ female, YKA male, ÇO female, ÇG female.
5. Experience and training	What experience or training did the researcher have?	ZYÖ previously worked as a family physician in a region with a dense geriatric patient population and in a palliative unit. ÇO has previously worked as a general practitioner with the elderly population within the scope of home care services. YKA has worked in geriatric studies. ÇG has worked with a large number of geriatric patients at a university family medicine clinic.
Relationship with participants		
6. Relationship established	Was a relationship established prior to study commencement?	ZYÖ administered the sociodemographic data form and questionnaire for the quantitative part of the study face-to-face while providing information for the qualitative part of the

		study (June 16, 2022-July 13, 2023).
7. Participant knowledge of the interviewer	What did the participants know about the researcher? e.g. personal goals, reasons for doing the research	The purpose of the study was summarised verbally, and the informed consent form was provided to the participants
8. Interviewer characteristics	What characteristics were reported about the interviewer/facilitator? e.g. Bias, assumptions, reasons, and interests in the research topic	Participants were familiar with the ZYÖ from the quantitative part of the study and knew that she works with older people
**Domain 2: Study design**		
Theoretical framework		
9. Methodological orientation and theory	What methodological orientation was stated to underpin the study? e.g. grounded theory, discourse analysis, ethnography, phenomenology, content analysis	Thematic analysis
Participant selection		
10. Sampling	How were participants selected? e.g. purposive, convenience, consecutive, snowball	Purposive sampling & maximum variance
11. Method of approach	How were participants approached? e.g. face-to-face, telephone, mail, email	Telephone
12. Sample size	How many participants were in the study?	8
13. Non-participation	How many people refused to participate or dropped out? Reasons?	Three participants refused to participate in the study. Their reasons are given in the text.
Setting		
14. Setting of data collection	Where was the data collected? e.g. home, clinic, workplace	Çukurova University, ZYO’s office
15. Presence of non-participants	Was anyone else present besides the participants and researchers?	No

16. Description of sample	What are the important characteristics of the sample? e.g. demographic data, date	Gender, age, education level/occupation, number of children, illness, and ageism survey scores
Data collection		
17. Interview guide	Were questions, prompts, and guides provided by the authors? Was it pilot-tested?	Yes, four semi-structured open-ended questions were prepared to reflect the participants' personal experiences regarding ageism. A pilot study was conducted for semi-structured questions
18. Repeat interviews	Were repeat interviews carried out? If yes, how many?	No
19. Audio/visual recording	Did the research use audio or visual recording to collect the data?	No
20. Field notes	Were field notes made during and/or after the interview or focus group?	No
21. Duration	What was the duration of the interviews or focus group?	Ranged between 5-11 mins
22. Data saturation	Was data saturation discussed?	Yes
23. Transcripts returned	Were transcripts returned to participants for comment and/or correction?	No
**Domain 3: Analyses and findings**		
Data analysis		
24. Number of data coders	How many data coders coded the data?	2
25. Description of the coding tree	Did the authors provide a description of the coding tree?	Yes
26. Derivation of themes	Were themes identified in advance or derived from the data?	Themes were derived from the data
27. Software	What software, if applicable, was used to manage the data?	ZYÖ transcribed the data verbatim into Word documents and grouped them in tables.
28. Participant checking	Did participants provide feedback on the findings?	No

Reporting		
29. Quotations presented	Were participant quotations presented to illustrate the themes/findings? Was each quotation identified? e.g. participant number	Yes
30. Data and findings consistent	Was there consistency between the data presented and the findings?	Yes
31. Clarity of major themes	Were major themes clearly presented in the findings?	Yes
32. Clarity of minor themes	Is there a description of diverse cases or a discussion of minor themes?	Yes

COREQ: Consolidated Criteria for Reporting Qualitative Research
