# Supplementary material for: Experiences of Ageism Among Older Adults Registered with a Family Health Centre: A Mixed-Methods Research Study
Source: Healthcare (Basel). 2026 Mar 21;14(6):801. doi: 10.3390/healthcare14060801 (PMC13026802; doi:10.3390/healthcare14060801)
Supplement: Supplementary file 1 [file healthcare-14-00801-s001.zip › File S3.pdf]

Supplementary File S3. Code Tree

Codes	Categories	Sub-themes	Themes
You're not like when you were young, you're done, it's not nice, it's not like it used to be, turning to ashes, you're past your prime	1) Negative thoughts about old age and their own aging	1) Age-related self stigma	1)Self-directed ageism
Illness, limitation of movement, health problems, weakness, inability to move, forgetfulness, my legs are swollen, health, tired quickly, strain, restricted social activity, fatigue, weakness, pain, insomnia, fatigue.	2) Discomfort and reduced participation in social activities	2) Physiology of old age and feeling restricted	2) Increasing health problems and social isolation with age
Being sidelined, not being listened to (by young people), not sitting next to us (by young people), ageism, disrespect (by young people), being looked down upon.	3) Social exclusion<br>4) Feeling of disrespect	3) Negative behaviors towards the elderly	3)Ageism
